# Nanocomposite Polysulfone/CB Modified by Melt Extrusion and Solution Mixing for Enhanced Removal of Uremic Toxins

**DOI:** 10.3390/ma18143352

**Published:** 2025-07-17

**Authors:** Marlene Andrade-Guel, Christian J. Cabello-Alvarado, Sendar Daniel Nery-Flores, Gregorio Cadenas-Pliego, Carlos Avila-Orta, Marissa Pérez-Alvarez, Diego Martínez-Carrillo, Zoe V. Quiñones-Jurado, Luis Cedeño Caero

**Affiliations:** 1 Center for Research in Applied Chemistry, Saltillo 25294, Coahuila, Mexico; gregorio.cadenas@ciqa.edu.mx (G.C.-P.); carlos.avila@ciqa.edu.mx (C.A.-O.); marissa.perez@ciqa.edu.mx (M.P.-A.); 2 Researcher for Mexico SECIHTI-CIQA, Av. Insurgentes Sur 1562, Col. Crédito Constructor, Alcaldía Benito Juárez, Mexico City 03940, Mexico; 3 Faculty of Chemical Sciences, Autonomous University of Coahuila, Saltillo 25280, Coahuila, Mexico; sendar.nery@uadec.edu.mx; 4 Center for Research in Applied Geosciences, Autonomous University of Coahuila, Nueva Rosita 26830, Coahuila, Mexico; diegomartinez@uadec.edu.mx; 5 Facultad de Ciencias Químicas, Universidad Juárez del Estado de Durango, Durango 34120, Durango, Mexico; zoevineth@gmail.com; 6 Catalysis Research Unit, Department of Chemical Engineering, Faculty of Chemistry, National Autonomous University of Mexico, Mexico City 04510, Mexico; caero@unam.mx

**Keywords:** polysulfone, carbon black, melt extrusion, urea, solution mixing

## Abstract

In this study, polysulfone-based nanocomposites with carbon black (CB) nanoparticles were fabricated to evaluate their urea-removal properties. The nanocomposites were obtained using two different methods: solution mixing and melt extrusion. These materials were evaluated using Fourier transform infrared spectroscopy (FTIR), which allowed for the identification of the corresponding functional groups within the polysulfone polymer matrix. X-ray diffraction (XRD) analysis was performed, confirming the amorphous structure of the polysulfone. The addition of modified carbon black shifted the most intense peak of the polysulfone. Thermogravimetric analysis (TGA) showed an increase in thermal stability with the addition of different concentrations of modified carbon black for solution-mixing method. Scanning electron microscopy (SEM) revealed that the melt-extrusion method presented a better dispersion of the nanoparticles, since large agglomerates were not observed. Additionally, a urea adsorption study was conducted, obtaining removal percentages of 76% and 72% for the extrusion and solution-mixing methods, respectively. It was demonstrated that the nanocomposite can be used for up to five cycles without losing urea-removal efficiency, whereas the efficiency of pure polysulfone decreases as the number of cycles increases. Finally, the hemolysis test was performed, and the nanocomposites showed less than 1% hemolysis, indicating that the material is non-hemolytic.

## 1. Introduction

Chronic kidney disease (CKD) affects more than 10% of the population worldwide. This condition stems from various chronic degenerative diseases, including diabetes mellitus and high blood pressure. Higher incidences have been documented in low- and middle-income countries due to high investment costs, infrastructure resources, and limited human resources. Mexico is one of the countries that currently does not have a database of CKD patients. It is estimated that around 14.5 million people suffered from CKD in 2017, and that same year, it was the second leading cause of death in the country [1,2,3].

There are two main treatments for CKD: peritoneal dialysis and hemodialysis. Hemodialysis was initially called “renal replacement therapy”. In recent years, synthetic membranes have been optimized to remove a higher percentage of uremic toxins and prevent protein loss [4].

Initially, natural membranes such as cellulose were selected for hemodialysis treatment because they could remove small water-soluble molecules, such as urea and creatinine; however, they did not remove medium-molecular-weight toxins and also had biocompatibility issues. As a result, synthetic membranes were developed to address the issues of biocompatibility and efficiency in toxin removal. They were made from synthetic polymers, including polyethersulfone, polymethylmethacrylate (PMMA), polyacrylonitrile, polyvinylidene fluoride, and polysulfone (PS) [5,6,7,8].

Polysulfone is one of the most important polymers in membrane manufacturing due to its unique properties, including resistance to acidic and basic environments, thermal stability, good mechanical properties, and optical transparency [9,10]. It can be easily processed and is a suitable candidate for applications such as gas separation, nanofiltration, drug delivery, hemodialysis, and other similar uses [11,12,13]. The preparation of polysulfone nanocomposites with carbonaceous nanostructures has been recently investigated due to the remarkable properties of these nanostructures, including their high surface area, conductivity, and mechanical properties. Among the carbon-based nanoparticles, the following stand out: single-walled nanotubes (SWNTs), multi-walled nanotubes (MWCNTs), graphene, nanofibers, and carbon black, among others [14]. 

Nechifor et al. developed new polymeric-nanotube composite materials based on polysulfone with different types of nanotubes—single-walled (SWNTs) and double-walled (DWNTs)—which were prepared in a phase inversion process by immersion precipitation, in which the polymer solution is immersed in a nonsolvent bath; the materials showed promising results in the adsorption of lead from a physiological liquid [15].

Pandele et al. [16] fabricated membranes by mixing polysulfone and reduced graphene oxide that was previously functionalized with 4-aminobenzo-15-crown-5 ether to remove metal ions in the hemodialysis process. The membranes were prepared through a phase inversion process. Furthermore, the incorporation of MWCNTs with polycitric acid and polyethersulfone (PES) using the wet–dry spinning technique has been studied to obtain hemodialysis membranes, evaluating these membranes for compatibility with the protein albumin. It is worth noting that commercial hemodialysis membranes, which are made of pure polysulfone, are not typically selective, retaining both toxins and proteins, the latter of which are essential for various human functions. The protein rejection results for the PES nanocomposite with a nanofiller based on polycitric acid-grafted-MWCNT obtained a rejection of 95.2%, while the pure PES membrane showed a value of 90.2% [17]. 

Another carbon structure with very interesting properties is carbon black; this structure stands out for its complex quasi-graphitic configuration and colloidal dimensions. Additionally, it has high purity due to its freedom from inorganic contaminants and organic residues, which are commonly found in most forms of carbon [18]. This carbon-based nanoparticle has been utilized as a nanofiller in various polymeric matrices, including low-density polyethylene, polyamide-6, polypropylene, and polysulfone, among others [19,20,21,22,23]. Khan et al. [24] fabricated a polysulfone-based membrane with carbon black chemically modified with ammonium persulfate. They used a phase inversion method for the preparation of the membranes. The results showed a membrane with excellent antifouling properties and good mechanical resistance, which was applied for water purification.

Based on the literature, this study aims to develop nanocomposites based on polysulfone and carbon black modified with citric acid for potential applications in the adsorption of uremic toxins. For this purpose, tests were carried out with two toxins (urea and creatinine), in addition to the hemolysis assay, to assess their effect on red blood cells. In our previous study, carbon black modified with citric acid was used to prepare a nanocomposite based on Nylon-6 for the adsorption of uric acid [25]. These new materials aim to enhance the properties of polysulfone, which is currently utilized as a membrane for hemodialysis.

## 2. Materials and Methods

### 2.1. Materials

The polymer used was Polysulfone U from M-2300-MR, a product of Solvay Specialty Polymers (Guangzhou, China). Carbon black VULCAN XC-72 CB with a diameter of 15 nm and a purity of 99% was used. The citric acid used has a purity of 99% (Sigma Aldrich, St. Louis, MO, USA). The creatinine (Sigma-Aldrich, ≥98%) and urea (Fagalab, Mocorito, Mexico, 99.4%) were used without prior treatment.

### 2.2. Modification of Carbon Black

The modification of carbon black with citric acid was carried out following the protocol of Andrade et al. [25], 2020. Carbon black was added to a concentrated citric acid aqueous solution at a 1:1 ratio. This process was facilitated by variable-frequency ultrasound energy, and the treatment was conducted for 60 min at room temperature. At the end of the reaction time, the samples were filtered and dried at 80 °C for 24 h.

### 2.3. Preparation of Nanocomposites

#### 2.3.1. Melt-Extrusion Process

**Figure 1:**
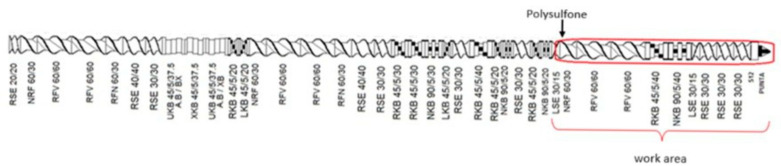
STEER Omega 30 model screw-extruder configuration.

Before extrusion, the polysulfone was dried in an oven at 120 °C for 12 h. First, a physical mixture of the polymer and nanoparticles was created at the concentrations considered for this study. Then, the polymer and nanoparticles were fed into the hopper. The extrusion process was carried out in a STEER Omega 30 twin-screw extruder (Bangalore, India), at a processing temperature of 330 °C, a pressure of 13 bar, and a speed of 100 rpm, using the screw configuration shown in Figure 1. As a post-extrusion system, a cooling bath was used at the outlet of the die, and pellets were obtained using a pelletizer.

#### 2.3.2. Mixing Solution

**Figure 2:**
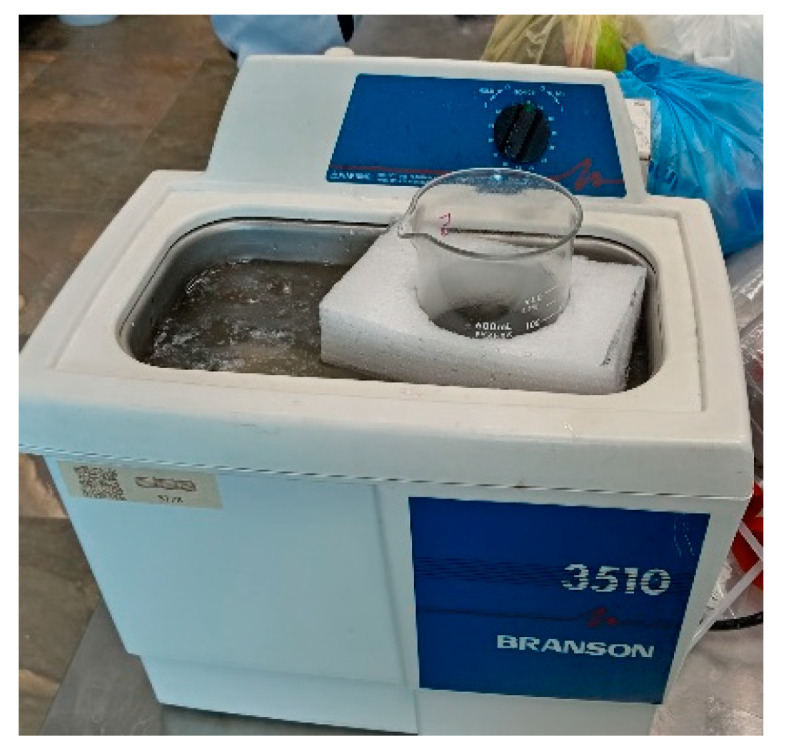
Mixing in solution by an ultrasonic bath. The nanocomposites obtained in the form of pellets by extrusion or film by mixing solution were ground and sieved through a 200-mesh sieve. For performing urea adsorption and hemolysis tests, the particle size was between 50 and 65 µm.

Polysulfone and modified carbon black films were prepared by solution mixing. The established concentration of carbon black particles was dispersed in 10 mL of chloroform in a Branson ultrasonic bath for 5 min (Figure 2). The polysulfone pellets were then dispersed in 40 mL of chloroform for 10 min. The carbon black solution was added to the polysulfone solution and subjected to 15 min of ultrasonication. After this time, the solution was allowed to dry at room temperature for 48 h, and a film was obtained. Table 1 shows identifications of the PSCB nanocomposites with different percentages of additives.

### 2.4. Characterization

The nanocomposites were characterized by FT-IR spectroscopy, using a Magna Nicolet 550 (Termo Fisher Scientific., Waltham, MA, USA). The pre-modified carbon black was dried in a vacuum oven at 100 °C for 15 h and then supported on KBr discs. The ATR module was used for the nanocomposites.

The thermogravimetric analysis was carried out using a TGA Q500 TA INSTRUMENTS Thermobalance (New Castle, PA, USA), establishing an initial temperature of 35 °C and a final temperature of 700 °C.

For the characterization by XRD, the X-RAY D8 ADVANCE ECO BRUKER diffractometer (Bruker, Billerica, MA, USA) was used, where the pellets or films were placed in the sample holder, and a scan of 10 to 80° was used. The morphology of the nanocomposite was observed using a JEOL JCM6000 scanning electron microscope (Jeol LTD., Akishima, Tokyo, Japan); the films were placed on a copper tape, and for the nanocomposites, the pellets were melted to form a film.

### 2.5. Urea Adsorption

The experimental process consisted of weighing 20 mg of the nanocomposite. Then, 50 mL of urea at a concentration of 390 mg/dL was added. The mixture was stirred at 100 rpm for 4 h, and 3 mL aliquots were taken every 15 min. The solutions were then read in a UV-Vis spectrophotometer at a wavelength of 210 nm. The test was triplicated. The removal percentage was calculated using the following equation, where *C_i_* is the initial concentration and *C_e_* is the final concentration:

(1) $$\%\;\mathrm{remove}=\frac{C_{i}-C_{e}}{C_{i}}\;\times \;100$$

### 2.6. Hemolysis Assay

The human blood sample was obtained from a healthy volunteer without any prior medical history of chronic conditions and without a history of ingesting any non-steroidal anti-inflammatory drug in the last 5 days. Phlebotomy was performed on the peripheral veins of the arm, using a Vacutainer^®^ 21G × 38 mm needle and a BD Vacutainer^®^ K2 EDTA tube (Aguascalientes, Mexico). After collection, the blood samples were centrifuged at 600× *g* for 5 min. A line was marked on the tube at the same height as the supernatant, which was then removed. The same volume of phosphate-buffered saline (PBS) solution was added to the erythrocyte suspension, mixed carefully, and centrifuged again at 600× *g* for 5 min. The supernatant was removed, and the washing process with PBS solution was repeated three more times. Once the washing steps were completed, the erythrocytes were suspended in PBS at a concentration of 2% (*v*/*v*). For this, 10 µL of the erythrocyte pellet was diluted in 480 µL of PBS with 10 µL of the nanoparticles dissolved in PBS at different concentrations (20, 10, 5 and 2.5 mg/mL) to obtain a final concentration in the microtube of 400, 200, 100 and 50 µg/mL. The samples were then incubated at 37 °C for 1h and were mixed every 15 min during the hour of incubation. After incubation, the samples were centrifuged at 2000× *g* for 5 min, and 100 µL of the supernatant was placed in one well of the 96-well microplate in triplicate. Finally, the samples were read at a wavelength of 540 nm using a spectrophotometer, EPOCH Biotek ELISA plate reader. PBS and distilled water were used as negative and positive controls, respectively. The equation used to calculate the percentage of hemolysis was as follows:

(2) $$\%\;\mathrm{hemolysis}=\left(\frac{{\mathrm{Sample}\;\mathrm{Abs}}_{540}\;-\;{\mathrm{Blank}\;\left(\mathrm{PBS}\right)\mathrm{Abs}}_{540}}{{\mathrm{Total}\;\mathrm{lysis}\;\left(dH_{2}O\right)\mathrm{Abs}}_{540}\;-\;{\mathrm{Blank}\;\left(\mathrm{PBS}\right)\mathrm{Abs}}_{540}}\right)\;\times \;100$$

## 3. Results and Discussions

### 3.1. Fourier-Transform Infrared Spectroscopy (FTIR)

**Figure 3:**
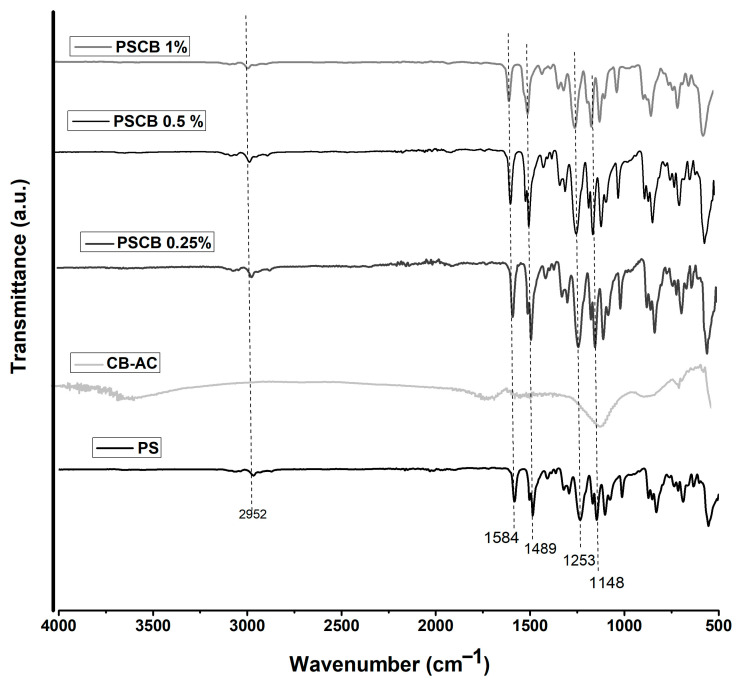
FT-IR spectra of PS neat, modified carbon black, and PSCB at different concentrations (0.25, 0.5 and 1%) by melt extrusion.

Figure 3 shows the FTIR spectrum of pure polysulfone and the polysulfone/carbon black nanocomposite produced by melt extrusion. The signal corresponding to pure polysulfone is present at 2966 cm^−1^; this small signal is associated with the symmetric stretching of -CH_3_. At 1484 cm^−1^ and 1583 cm^−1^, the characteristic signals of the aromatic rings of polysulfone are found; at 1236 cm^−1^ and 1147 cm^−1^, bands of asymmetric and symmetric stretching of the sulfone group are shown, and a signal can also be observed at 1012 cm^−1^ due to the presence of an aromatic compound. If we compare the FTIR spectra of the nanocomposite samples at concentrations of 1% and 0.5% of carbon black against the spectrum of pure polysulfone, a similarity in the spectra can be seen.

Sun et al. in 2018 synthesized a polysulfone/activated carbon nanocomposite doped with silver nanoparticles [26]. In the FTIR characterization, they found that their four samples had obtained the characteristic signals of polysulfone: the C-H stretching vibration peaks appeared at 2969 cm^−1^; the benzene ring vibration peaks appeared between 1600 cm^−1^ and 1470 cm^−1^; and the asymmetric C–O stretching signals appeared at 1252 and 1016 cm^−1^. Since they did not find signals for the activated carbon doped with nano-silver, they concluded that this was because it was homogeneously mixed in the polysulfone matrix.

**Figure 4:**
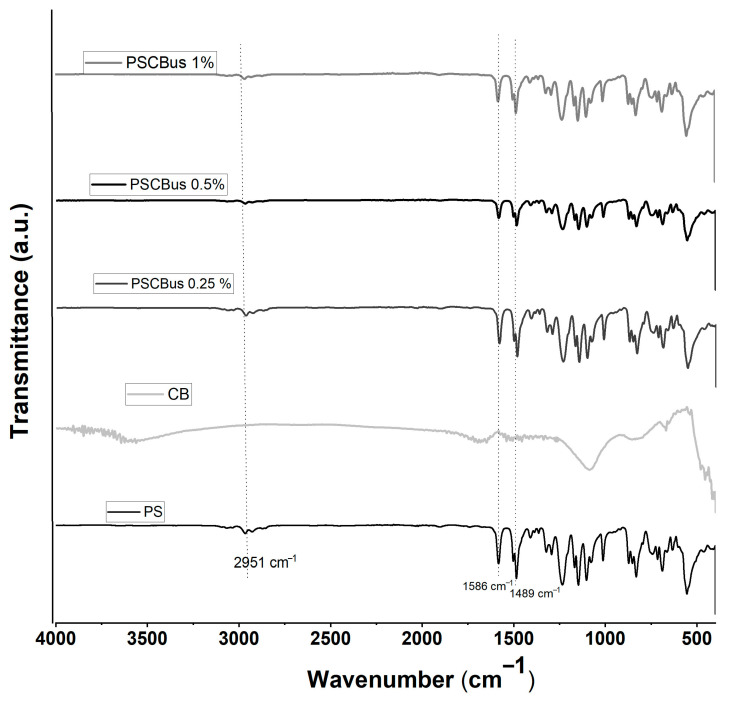
FT-IR spectra of PS neat and PSCBus at different concentrations (0.25, 0.5 and 1%) by mixing in solution.

Figure 4 shows the infrared spectra of PSCBus nanocomposites made by the solution-mixed method using an ultrasonic bath. The characteristic bands of the polysulfone polymer matrix can be seen at 2951 cm^−1^, corresponding to the stretching of CH_3_-; at 1489 and 1586 cm^−1^, there are signals attributed to the aromatic rings that are part of the polysulfone structure. This finding aligns with the report by Daramola et al. in 2017 [27]. Due to the carbon black loading, where the maximum is 1% by weight, no characteristic signals of carbon black are detected; this has already been reported by other authors [25].

### 3.2. X-Ray Diffraction (XRD)

**Figure 5:**
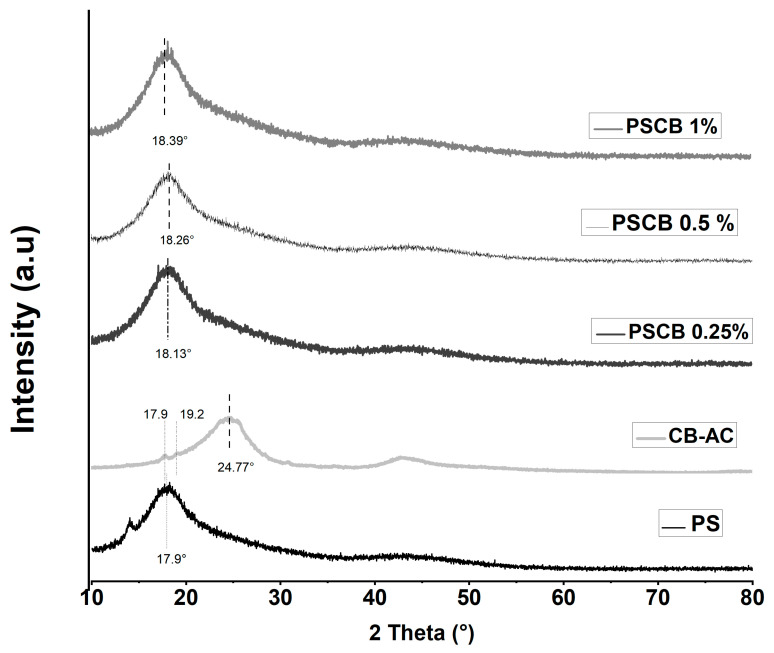
XRD patterns of neat PS, CB-AC, and PSCB nanocomposites with 0.25%, 0.5%, and 1% by melt extrusion.

X-ray diffraction patterns for PSCB nanocomposites at different concentrations, obtained by melt extrusion, for polysulfone and modified carbon black, are shown in Figure 5. The diffraction peak of polysulfone is broad and centered at 17.9°, indicating a quasi-amorphous structure [28]. Citric acid-modified carbon black presents a peak at 2θ = 24.8° assigned to the carbon black structure, but two additional signals are also observed at 2θ = 17.9° and 2θ = 19.2°, which are attributed to the citric acid modification. Regarding the diffraction patterns of polysulfone with modified carbon black at different concentrations, all exhibit a broad peak around 2θ = 18.0°, which is attributed to a change in the polysulfone structure. The diffraction peak of carbon black is not observed, suggesting a good dispersion of carbon black in the polymer matrix. This finding is consistent with the report by Bouchereb et al. for polysulfone/graphene oxide-silver nanocomposites [29].

**Figure 6:**
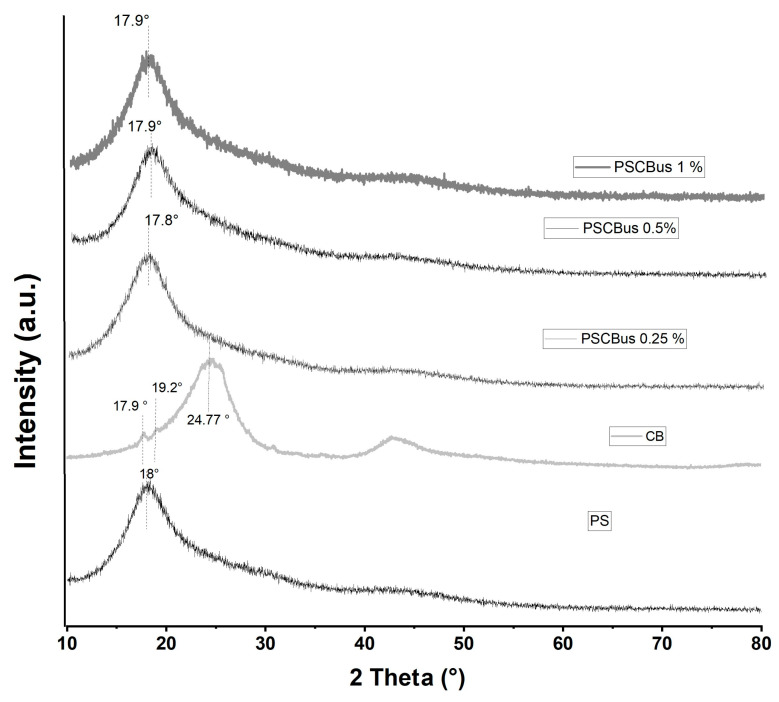
X-ray diffraction patterns of PS neat and PSCBus at different concentrations (0.25, 0.5 and 1%) by mixing in solution.

Figure 6 shows the diffraction patterns of neat polysulfone, carbon black, and polysulfone–carbon black films at three different concentrations—0.25%, 0.5%, and 1%—using solution mixing. Polysulfone appears as an amorphous structure because it only has a peak at 18.0°. The films of polysulfone and carbon black nanocomposites exhibited a single amorphous peak at 17.8–17.9°. No characteristic peaks were observed with carbon black; the same was observed using the melt-extrusion method. Other authors have reported that in the preparation of nanocomposites and polysulfone with carbon nanostructures, no characteristic peak of the carbon nanostructure appears; they attribute this to the good dispersion of the nanoparticles in the polymer matrix and the low concentration of carbon nanoparticles [29,30]. 

### 3.3. Thermogravimetric Analysis (TGA)

**Figure 7:**
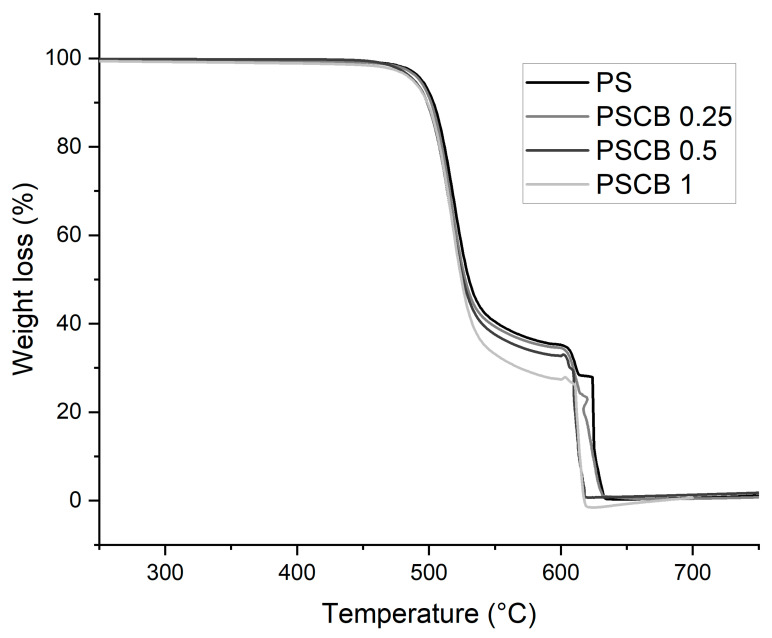
Thermogravimetric analysis of PS neat and PSCB at different concentrations (0.25, 0.5, and 1%).

The thermogram shown in Figure 7 corresponds to the nanocomposites obtained by melt extrusion, where a first weight loss is observed between 470 °C and 603 °C for all three samples. The second loss occurs from 630 °C to 620 °C; both losses are attributed to the thermal decomposition of the polymeric matrix. The results show that increasing the load from 0.5% decreases the thermal stability. In this method for obtaining polymeric nanocomposites, carbon black was thoroughly mixed. It should be noted that these particles possess good thermal conductivity properties, which increases heat in specific regions and accelerates degradation. Some research notes that when incorporated into a polymer matrix, they can interconnect with the polymer and cause an increase in thermal conductivity, thereby decreasing their thermal stability [31].

The thermal degradation temperatures for PS and PSCB 0.25% were 634 °C. For the 0.5% and 1% nanocomposites, they are 620 °C and 619 °C, respectively. Nayak et al. [32] reported that adding 3% wt carbon nanofibers to a polysulfone matrix via solution mixing with an ultrasonic horn improved thermal degradation stability—a behavior attributed to the barrier effect of the carbon nanofibers.

In the present study, using an ultrasonic bath, the thermal stability of the nanocomposites decreased, which can be attributed to the low dispersion of carbon black in the composites obtained by solution.

**Figure 8:**
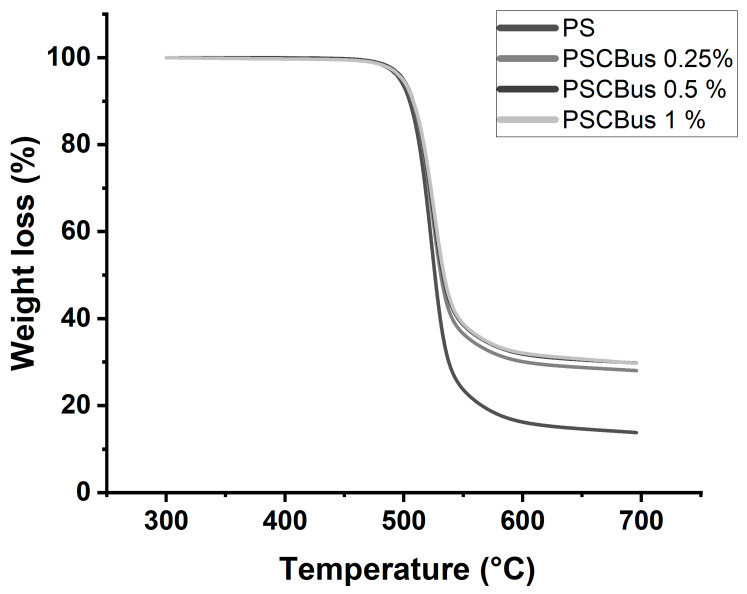
Thermogravimetric analysis of PS neat and PSCBus at different concentrations (0.25, 0.5 and 1%) by mixing in solution.

The TGA results of the polysulfone nanocomposites obtained by the solution-mixing method are presented in Figure 8. Pure polysulfone has a degradation temperature that starts at 533 °C. After the addition of modified carbon black at a loading of 0.25%, the degradation temperature is 542 °C. The PSCB 0.5% and PSCB 1% nanocomposites both present a degradation temperature of 546 °C. The thermal stability of the polymer is improved after adding the modified carbon black; this can be attributed to the formation of hydrogen bonds and electrostatic interactions between the polymer matrix and the carbon black modified with carboxylic acids [33]. The carbon black is homogeneously dispersed in the polymer matrix; as a result, it prevents thermal transfer and causes instability in the nanocomposite. This has already been reported previously for nanocomposites based on polysulfone and carbon nanotubes (CNTs) prepared by solution mixing [34]. 

### 3.4. Scanning Electron Microscopy (SEM)

**Figure 9:**
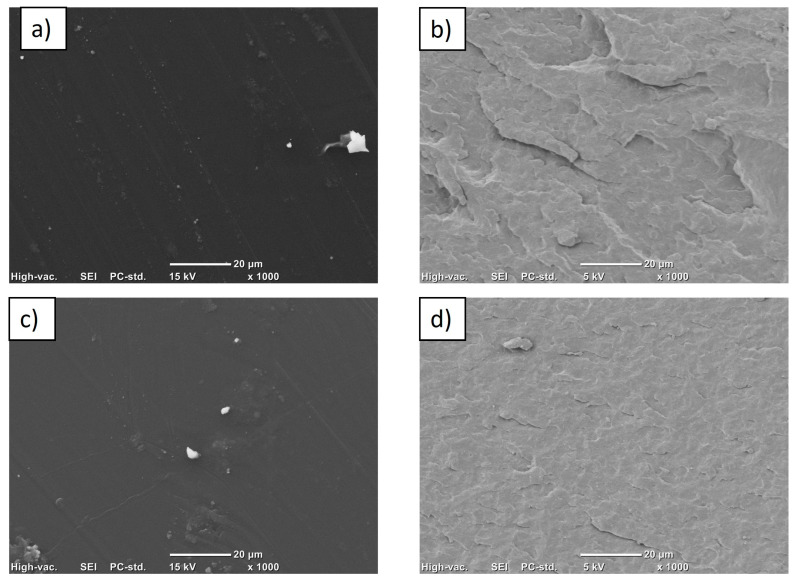
SEM images of nanocomposite of (**a**) PSCB us 0.5%, (**b**) PSCB 0.5%, (**c**) PSCB us 1%, and (**d**) PSCB 1%.

SEM images of carbon black nanocomposites in polysulfone matrix by the solution-mixing method are shown in Figure 9a,c. As can be seen, there are few large agglomerates and the film area is homogeneous, so the nanoparticles were well-integrated into the matrix; this has already been reported for polysulfone composites and different graphite structures [35]. The same figure shows micrographs of the nanocomposites obtained by extrusion Figure 9b,d. In both images, the surface of the homogeneous matrix is observed, without the presence of large agglomerates; only in the PSCB 1% nanocomposite is a large agglomerate observed in the upper part, indicating that the melt-extrusion process ensures a good dispersion of the nanoparticles in the polymeric matrix. However, some agglomerates probably remained.

### 3.5. Urea Adsorption

**Figure 10:**
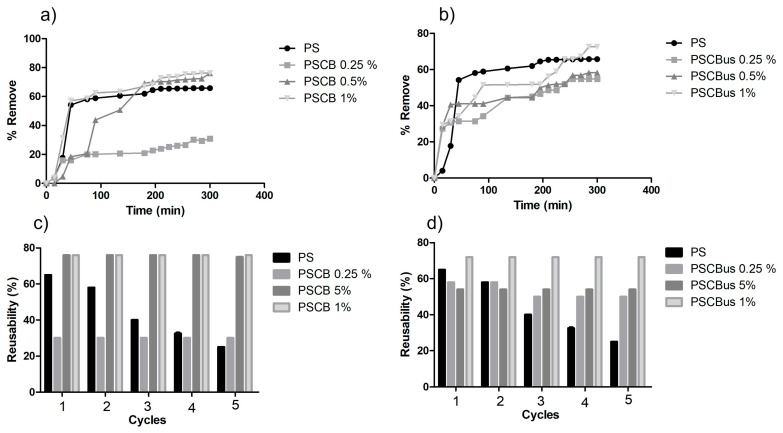
(**a**) Effect of contact time on the removal percentage of urea by nanocomposites PSCB, (**b**) effect of contact time on the removal percentage of urea by PSCBus, (**c**) Reusability of nanocomposites PSCB in five consecutive cycles, and (**d**) Reusability of nanocomposites PSCBus in five consecutive cycles.

UV-Vis adsorption tests were performed on pure polysulfone and the polysulfone/carbon black nanocomposite at different concentrations (0.25%, 0.5%, and 1%) obtained by two different methods: melt extrusion and solution mixing. Pure polysulfone exhibited an adsorption percentage of 65% for urea after 4 h, which agrees with the study by Zailani et al. [36], which reported 66% adsorption. The results of the removal percentage of the nanocomposites obtained by melt extrusion are shown in Figure 10a, where it can be observed that the PSCB 1% and PSCB 0.5% nanocomposites had the highest adsorption for urea, where both obtained 76% removal. In another study by our working group, a 90% urea-removal percentage was reported using Nylon-6 as a matrix and a synergy of two carbon black nanoparticles and graphene nanoplatelets [37]. Figure 10c shows the reusability test, which shows that after the fifth cycle of use, the polysulfone lowers its urea-removal percentage; from the second cycle onwards, a decrease is observed; however, in the PSCB at different concentrations, it maintains the same removal value after five cycles. This indicates that the material can be reused, which would help reduce the costs associated with the material for hemodialysis. 

On the other hand, in Figure 10b, the results of the urea-removal percentage of the nanocomposites obtained by solution mixing using ultrasound are presented. PSCBus 0.25% and PSCBus 0.5% obtained 54% and 58%. However, when the concentration of the nanoparticles increased by 1%, a urea-removal percentage of 72% was obtained. A reusability study was also conducted, where the same behavior observed in the nanocomposites by extrusion is noted. Specifically, a decrease in the percentage of urea removal is observed in pure polysulfone as the number of cycles increases. At the same time, the PSCBus nanocomposites maintain the same percentage obtained from the first cycle.

Comparing the values, we can conclude that adding carbon black nanoparticles increases the urea-removal percentage compared to pure polysulfone. Regarding the production methods, the difference is slight in the case of adsorption. In this case, the nanocomposite with the best urea adsorption properties would be the nanocomposite obtained by extrusion. Therefore, the polysulfone/carbon black nanocomposite can be used in hemodialysis treatment.

### 3.6. Hemolysis Test

**Figure 11:**
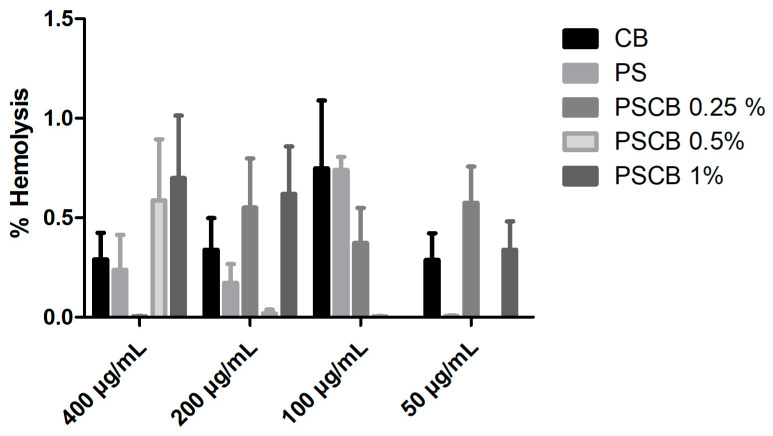
A bar graph shows the percentage hemolysis of different powders with varying concentrations for CB, PS, and nanocomposite PSCB at different concentrations (0.25%, 0.5%, and 1%).

For the hemolysis study, samples obtained using the melt-extrusion method were chosen due to their high percentage of urea removal. It is worth noting that this method can be scaled to an industrial level, does not require solvents, and is environmentally friendly, as it does not generate byproducts. One way to ensure the safety of new biomaterials that come into direct contact with blood is to study them to verify that they are non-toxic to the human body. Figure 11 presents the results of the hemolysis percentage of the pure polysulfone polymer matrix, carbon black, and PSCB nanocomposites at different concentrations. All samples were below 1% hemolysis; for a material to be considered toxic or hemolytic, it must obtain a percentage above 5%. Therefore, these nanocomposites are non-toxic and could be regarded as hemocompatible, since they do not break apart red blood cells. The concentrations used were 50 to 400 µg/mL; the results at 400 µg/mL stand out, where it is observed that PS, CB, and PSCB 0.25% obtained percentages below 0.5%. In one study, the effect of placing carbon black in contact with platelets and erythrocytes was evaluated, investigating its impact on the membranes of these cells after 24 h of contact [38]. The results obtained showed that there are no changes in the morphology of erythrocytes and platelets.

## 4. Conclusions

In this work, nanocomposites based on polysulfone and carbon black modified with citric acid were prepared using two different methods: melt extrusion and solution mixing with an ultrasonic bath. The results of infrared spectroscopy portray the main signals of the polymeric matrix. The presence of the functional groups of carbon black was not detected due to the low load and a homogeneous mixture of the nanocomposite, or because the signals overlap those of carbon black.

Using X-ray diffraction, an amorphous matrix of polysulfone was detected, and shifts in the diffraction peak of the polysulfone were observed using both methods. The nanocomposites obtained by solution mixing improved the thermal stability of the polysulfone. The dispersion of carbon black in the polymeric matrix was homogeneous, with few agglomerates detected, indicating good dispersion by both methods. Urea-removal rates of 76% and 72% were obtained for the sample containing 1% carbon black using the melt-extrusion and solution-mixing methods, respectively. This nanocomposite can be used for up to five cycles, making this material reusable for hemodialysis treatment. It was also found to be non-toxic and considered hemocompatible.

## Figures and Tables

**Table 1:** Identification of the PSCB nanocomposites with different percentages of additives (0.25, 0.5 and 1%).

Scheme	Method	Description
PS	Extrusion and mix solution	Pristine PS
CB-AC	Extrusion and mix solution	Carbon black
PSCB-0.25%	Extrusion	PS with 0.25% of carbon black modified with citric acid
PSCB-0.5%	Extrusion	PS with 0.5% of carbon black modified with citric acid
PSCB-1%	Extrusion	PS with 1% of carbon black modified with citric acid
PSCBus-0.25%	Ultrasound	PS with 0.25% of carbon black modified with citric acid
PSCBus-0.5%	Ultrasound	PS with 0.5% of carbon black modified with citric acid
PSCBus-1%	Ultrasound	PS with 1% of carbon black modified with citric acid

## Data Availability

The original contributions presented in this study are included in the article. Further inquiries can be directed to the corresponding authors.
